# Support for Social Change Among Members of Advantaged Groups: The Role of a Dual Identity Representation and Accepting Intergroup Contact

**DOI:** 10.1177/01461672221086380

**Published:** 2022-04-28

**Authors:** Lisa Katharina Frisch, Simone Sebben, Luisa Liekefett, Nurit Shnabel, Emilio Paolo Visintin, Johannes Ullrich, Tabea Hässler

**Affiliations:** 1 University of Zurich, Switzerland; 2 Osnabrück University, Germany; 3 Tel Aviv University, Israel; 4 University of Ferrara, Italy; 5 University College Dublin, Ireland

**Keywords:** common-ingroup identity model, needs-based model, advantaged group members, collective action, social change

## Abstract

This preregistered research analyzed survey data from ethnic and religious advantaged groups in 12 countries (*N =* 2,304) to examine the interplay between two determinants of support for social change toward intergroup equality. Drawing on the needs-based model and the common-ingroup identity model, we hypothesized that the experience of accepting intergroup contact and the endorsement of a dual identity representation of intergroup relations would be associated with greater support for equality. Furthermore, integrating the logic of both models, we tested the novel hypothesis that the positive effect of accepting contact on support for equality would be stronger under a high (vs. low) dual identity representation. While the predicted main effects received empirical support, we found no evidence for the expected interaction. These findings suggest that interventions to foster support for social change among advantaged group members can promote accepting contact and a dual identity representation independently of each other.

When do the advantaged stand up for intergroup equality? What makes it more likely for them to actively support actions and policies that aim to establish social justice? Allport’s (1954) seminal contact hypothesis predicted that if members of advantaged groups experienced positive interactions with members of disadvantaged groups, they would abandon their prejudice. The hope was that prejudice reduction would ultimately lead to greater support for equality. While the notion that intergroup contact leads to prejudice reduction has received extensive empirical support (see Pettigrew & Tropp, 2006), the assumption that prejudice reduction would lead to support for equality has been challenged by findings that advantaged group members’ friendly relations with disadvantaged group members do not necessarily translate into greater support for equality (Jackman & Crane, 1986; see also Dixon & Levine, 2012). The processes involved in advantaged group members’ support for equality may be more complex than initially assumed.

Identifying the conditions under which advantaged group members support social change toward equality has critical practical importance. Although research on support for social change has traditionally focused on what motivates disadvantaged group members to act collectively to improve their conditions (see Wright, 2010), intergroup equality is plausibly reached through a joint effort *across* disadvantaged and advantaged groups. The participation of advantaged group members in support for social change plays a crucial role in shifting public opinion toward the rights of the disadvantaged, and thus facilitates lasting social equality (Radke et al., 2020). As a step toward addressing the existing lacuna in the literature on this topic, both Radke et al. (2020) and Craig et al. (2020) have recently proposed organizing frameworks for understanding the conditions and motivations underlying advantaged group members’ support for social change. Both frameworks stress social identification processes as a critical factor determining advantaged group members’ support for social change. This factor may manifest in advantaged group members’ identification with a shared politicized identity (e.g., as feminists or anti-racist, Radke et al., 2020; see also Subašić et al., 2008) as well as with a shared stigmatized identity (Craig et al., 2020), which may arise as a result of similar experiences of discrimination—albeit in different contexts (e.g., as members of sexual vs. racial minorities).

The present research aimed to contribute to the emerging effort to understand the factors influencing advantaged group members’ support for social change. It builds on the insights that identity-related processes play a pivotal role in explaining advantaged group members’ support for change (Craig et al., 2020; Radke et al., 2020), and that positive intergroup contact can affect these processes and consequent support for change (e.g., see Hässler et al., 2020; Reimer et al., 2017; Selvanathan et al., 2018, for the positive association between intergroup contact and advantaged group members’ support for change). Based on these insights, we draw on two theoretical models that examine the link between advantaged group members’ social identity and their attitudes toward disadvantaged groups: the needs-based model (Shnabel & Nadler, 2015) and the common-ingroup identity model (Gaertner & Dovidio, 2000).

Following the needs-based model, we examined whether the experience of moral acceptance during intergroup contact (defined as interpersonal interactions in which “advantaged group members feel welcomed and perceived as moral by disadvantaged group members”; Hässler et al., 2021, p. 4) would be associated with advantaged group members’ greater support for social change. Following the common-ingroup identity model, we examined whether advantaged group members who endorse a dual identity representation of intergroup relations—in which the advantaged and disadvantaged groups are viewed as two separate subgroups within a common, superordinate group—would also be more supportive of social change. Finally, integrating the logic of the two models, we discuss whether our data support the existence of an interaction effect, such that the positive effect of morally accepting intergroup contact on advantaged group members’ support for change would be stronger under a high (vs. low) dual identity representation.

## The Needs-Based Model

The needs-based model posits that, in societies that formally endorse egalitarian values, social inequalities asymmetrically threaten advantaged and disadvantaged groups’ positive social identities (Shnabel & Nadler, 2015). Whereas disadvantaged groups experience a threat to their identity as agentic and competent (e.g., Fiske et al., 2002), advantaged groups, who are viewed as benefiting from unearned privileges (Vorauer et al., 1998) and are often stereotyped as cold and bigoted (e.g., Cuddy et al., 2008), experience a threat to their ingroup’s moral identity. Because group members are generally motivated to maintain a positive social identity (Tajfel & Turner, 1979), advantaged group members often seek moral-social acceptance from disadvantaged group members (Aydin et al., 2019; Hässler et al., 2019; Siem et al., 2013). Illustrating this motivation, within interracial (but not intraracial) interactions, White Americans primarily wished to be liked and perceived as nonracist (rather than to be respected and perceived as competent) by Black Americans (Bergsieker et al., 2010).

At first glance, it might seem counterintuitive that advantaged group members care about being morally accepted by a disadvantaged group, which has a lower status and less power and influence than their ingroup (Vorauer & Sakamoto, 2008). However, when inequality is perceived as illegitimate, disadvantaged group members are perceived as victims of the advantaged group (Moscovici & Pérez, 2009), whose members seek forgiveness and moral affirmation from those whom they perceive as their ingroup’s victims. Moreover, with decreasing perceived legitimacy of the group status difference, disadvantaged group members are seen as better judges of moral goodness, and thus advantaged group members become invested in their moral evaluations (Vorauer, 2006).

In line with these considerations, moral-social acceptance by the disadvantaged group has been shown to improve advantaged group members’ attitudes toward the disadvantaged group and their support for social change toward greater equality (e.g., Shnabel et al., 2013). To illustrate, a recent multinational survey showed that cis-heterosexual individuals/ethnic majority members (representing socially advantaged groups) were more supportive of social change toward greater equality (e.g., willing to participate in demonstrations or support equality-promoting policies such as same-sex marriage or racial affirmative action) when they experienced their contact with sexual and gender minorities/ethnic minorities (representing socially disadvantaged groups) as morally accepting (Hässler et al., 2021). These findings demonstrate that the satisfaction of advantaged group members’ need for moral-social acceptance does not result in moral licensing effects (Merritt et al., 2010), in which social inequalities are legitimized. Instead, disadvantaged group members’ readiness to satisfy advantaged group members’ needs and help them restore their positive moral identity increased advantaged group members’ readiness to reciprocate by supporting social change toward greater equality, even at the cost of relinquishing power and advantage (see also SimanTov-Nachlieli et al., 2018).

However, for this reciprocal process to be successful, the model assumes that the advantaged group needs to perceive the disadvantaged group as belonging to the same moral community (Tavuchis, 1991), sharing common values, norms, and moral standards and rights (Shnabel & Ullrich, 2013). The more both groups are perceived to belong to the same moral community (see Opotow, 1990), the more are advantaged group members likely to experience a threat to, and a consequent need to restore, their moral identity. This means that identity representations (e.g., the degree to which advantaged group members perceive the disadvantaged group not only as distinct but also as sharing a common superordinate identity) should influence the extent to which moral-social acceptance by the disadvantaged would indeed foster support for intergroup equality among the advantaged. To understand this influence, we drew on the common-ingroup identity model.

## The Common-Ingroup Identity Model

Categorizing people into ingroup and outgroup members leads to intergroup bias, such that ingroup members are systematically favored over outgroup members (Tajfel & Turner, 1979; Turner et al., 1987; see Dovidio & Gaertner, 2010, for a review). Grounded in this notion, the common-ingroup identity model suggests that how people cognitively represent their ingroup and relevant outgroups shape intergroup relations (Gaertner & Dovidio, 2000, 2012). The model differentiates between four distinct identity representations. The “separate individuals” representation means de-categorization, in which people are viewed as differentiated individuals who do not belong to any group(s). Almost by definition, such a representation masks the need for any group-based social change (Dovidio et al., 2009). The other three representations (i.e., separate, common-ingroup, and dual identity), by contrast, involve categorizations at the group level.

Perceiving the ingroup and the outgroup as utterly separate groups (i.e., a “separate identity” representation) may pose obstacles to advantaged group members’ support for social change on behalf of disadvantaged groups. Indeed, perceiving a sharp distinction between groups (“us” vs. “them”) has been shown to increase ingroup favoritism and outgroup negativity, which impede positive intergroup relations (Dovidio & Gaertner, 2010).

A “common-ingroup” representation means perceiving both groups as sharing a superordinate identity. For instance, if Swiss nationals and immigrants living in Switzerland identify themselves as being all inhabitants of the same country, Switzerland, they hold a common-ingroup identity as Swiss. This representation, which emphasizes commonalities between groups, thereby extending ingroup favoritism to (former) outgroup members (Gaertner & Dovidio, 2000), has been shown to reduce intergroup prejudice (e.g., Kunst et al., 2015), improve intergroup attitudes (e.g., Vezzali et al., 2015), and increase intergroup cooperation (Beaton et al., 2012; see Gaertner & Dovidio, 2012, for a review). Regarding support for social change toward greater equality, however, the common-ingroup identity has its caveat: The emphasis on commonalities masks disparities between groups (Banfield & Dovidio, 2013), thereby perpetuating social inequalities by undermining group members’ willingness to support change (see also the “irony of harmony” effect, Saguy et al., 2009).

Finally, it is possible to share a common-ingroup identity without giving up subgroup identities (a “dual identity” representation, Dovidio et al., 2009). Thus, group members can perceive themselves as belonging to two separate groups within one common, superordinate group. For instance, Swiss nationals and immigrants living in Switzerland might categorize themselves in terms of a dual identity, if both are aware of their different origins, while also acknowledging their common identity as Swiss. A dual identity emphasizes commonalities, but at the same time discloses subgroup disparities such that inequalities are not swept under the proverbial carpet (Dovidio et al., 2004, 2016). Consequently, a dual identity representation is most successful in fostering support for social change among advantaged group members as compared to the remaining identity representations (Banfield & Dovidio, 2013; see also Hornsey & Hogg, 2000, for the positive effect of a dual identity in reducing intergroup bias).

## Integrating the Needs-Based Model and the Common-Ingroup Identity Model

Integrating the logic of the needs-based model and the common-ingroup identity model gives rise to novel predictions about advantaged group members’ support for social change. Specifically, denying that group categories exist, as with a “separate individuals” representation, eliminates the threat to advantaged group members’ moral identity (see Leach et al., 2001), which should reduce the effect of moral-social acceptance on advantaged members’ support for change. In a similar vein, when the disadvantaged group is perceived as separate, its moral-social acceptance might not be psychologically meaningful for advantaged group members because they do not see the two groups as belonging to the same moral community (Shnabel & Ullrich, 2013). Thus, the effect of moral-social acceptance on advantaged members’ support for equality should be relatively small (if any). Furthermore, when the disadvantaged and the advantaged groups share a common-ingroup identity, the threat to the moral identity of the advantaged group is removed (because the focus on commonalities distracts from existing inequalities between groups), eliminating advantaged group members’ need for moral-social acceptance. Thus, again, moral-social acceptance should have relatively little effect on advantaged members’ support for equality. Under a dual identity representation, however, the common superordinate group identity causes advantaged group members to care about disadvantaged group members’ moral-social acceptance, while the subgroup identities maintain the awareness of group-based inequalities. Therefore, the effect of receiving moral-social acceptance from the disadvantaged on advantaged group members’ support for intergroup equality is likely to be stronger.

Informed by both models and their theoretical integration, we hypothesized the following:

**Hypothesis 1 (H1):** Accepting intergroup contact should be positively related with support for social change (confirming the findings of Hässler et al., 2021).
**Hypothesis 2 (H2):** A dual identity representation should be positively related with support for social change (replicating the findings of Banfield & Dovidio, 2013).
**Hypothesis 3 (H3):** The positive relation of accepting contact and support for social change should be stronger when the dual identity representation of advantaged group members is high rather than low (a novel hypothesis and tested here for the first time).

## The Present Research

We tested our preregistered hypotheses using survey data collected by the Zurich Intergroup Project (ZIP, Hässler et al., 2020). In 12 countries, advantaged groups were identified in terms of their numeric size, as well as their relative status and power within each country (cf. Seyranian et al., 2008). These included ethnic or racial groups (e.g., Whites in Brazil), religious groups (e.g., Non-Muslims in Germany), or members of host societies (i.e., country nationals, for example, Swiss nationals in Switzerland). Disadvantaged outgroups instead represented refugee and immigrant minorities (e.g., Portuguese immigrants in Switzerland), ethnic or racial minorities (e.g., Blacks in Brazil), and religious minorities (e.g., Muslims in Germany). For the present research, members of advantaged groups indicated (a) their contact experiences with structurally disadvantaged groups, (b) the extent to which they represented groups in terms of a dual identity, and (c) their support for social change. The preregistration of hypotheses, sample, study design, and analyses, along with the data, analysis scripts, materials (including a full disclosure of measures in the ZIP codebook), and an online appendix is available at https://osf.io/teb8g/. We report all data exclusions and results of preregistered hypotheses and corresponding analyses in the main text.

As detailed in the preregistration, the key dependent variable was past behavior in support of social change. Specifically, while participants who took part in the ZIP completed several measures of support for social change, we reasoned that this particular measure is most suitable for the current study because it asked respondents about their *actual past behavior* supporting change (e.g., attendance of meetings and workshops) rather than about their future behavioral intentions (e.g., willingness to attend meetings and workshops). So, using this measure, our study accounts for the possibility of a gap between people’s behavioral intentions and actual behavior (e.g., Webb & Sheeran, 2006). As such, our study extends prior research, which has typically assessed participants’ intentions for (instead of past behavior of) support for social change (e.g., Banfield & Dovidio, 2013). Nevertheless, to make our findings more comparable to prior research, we also explored the effects of accepting contact and dual identity on intended support for social change.^1^

In addition, to isolate the effects that were the focus of the present research, we explored the results while using covariates. First, to examine the unique effect of morally accepting contact and to differentiate it from the simple experience of pleasant intergroup interactions, we controlled for positive contact (which has been shown to strongly predict advantaged group members’ greater support for change; e.g., Hässler et al., 2020). Second, to examine the unique effect of a dual identity representation and differentiate it from the three remaining identity representations (common-ingroup identity, separate identity, and separate individuals), which could be potentially overlapping (i.e., to endorse a dual identity representation, one may need to endorse both a common-ingroup identity and separate identity representations), we used the three items assessing these identity representations as covariates. Finally, to enhance the robustness of our findings and conclusions, we used both conventional metric analyses and ordinal Bayesian regression models. Table 1 summarizes the terms we used for the different regression models.

**Table 1.** Model Terms Used in the Regression Analyses.

Analysis	Model
Core model	Past/Intended support = Accepting contact × Dual identity
Controlling for positive contact and remaining identity representations	Past/Intended support = Accepting contact × Dual identity × Positive contact × Common-ingroup identity × Separate identities × Separate individuals
Ordinal Bayesian analyses	Past/Intended support = Accepting contact × Dual identity Past/Intended support = Accepting contact + Dual identity

*Note.* We ran each model for past support and intended support separately. The symbol × indicates that all main effects and two-way interactions were tested. The symbol + indicates that only main effects were tested. Additional models (e.g., with demographic and psychological indicators as covariates; see below) are reported in the online appendix.

### Method

#### Participants

As determined a priori in the preregistration, we used a subsample of members of ethnic, racial, and religious advantaged groups, *N* = 4,105, from the ZIP data set (Hässler et al., 2020; the online appendix provides full information on the selection and characteristics of subsamples and their sizes). All participants provided informed consent. According to the preregistered criteria, we excluded participants who failed one or both attention checks (e.g., “When you have read this item, please select the second point on the scale [to the right of ‘Strongly disagree’]”; *n* = 655), and who had more than 20% missing values^2^ on the items used in this article (*n* = 903). We did not include samples that were collected after the main wave of data collection (*n* = 90), to keep samples as homogeneous as possible. Deviating from our preregistration, we decided to exclude the sample of U.S. Americans reporting their relationship with Blacks (*n* = 153) because a different scale (sliders instead of Likert-type scales) was used to assess support for social change. These exclusions (*n* = 1,801 overall) did not meaningfully impact the statistical conclusions (for details, see online appendix).

The final sample size was *N* = 2,304 (1,521 women, 773 men, and 10 other, *Mdn_age_* = 24 years, range = 16–81 years), and it contained 20 subsamples of ethnic, racial, and religious advantaged groups. A sensitivity analysis for a 5% level of significance and a power of 95% revealed that this sample size was sufficient to detect effects as small as *f*^2^ = 0.0007.

#### Measures

##### Accepting contact

Two items measured accepting contact^3^ with disadvantaged groups (see Hässler et al., 2021): “I felt welcomed and accepted by [disadvantaged group members] with whom I had contact” and “I felt that [disadvantaged group members] with whom I had contact saw me as prejudiced or immoral” (reverse coded). Scales ranged from 1 = *Strongly disagree* to 7 = *Strongly agree* (see Data Preparation section for reliabilities).

##### Identity representations

Four items (as in González & Brown, 2006) assessed participants’ endorsement of the four identity representations. Specifically, on scales from 1 = *Not at all* to 7 = *Very much*, participants indicated “To what extent do you view the [advantaged group] and the [disadvantaged group] as . . . one common group / two separate groups / two separate groups within one common group / all unique individuals?”

##### Past support for social change

Table 2 shows the six items used to assess past behavior in support of social change (adapted from Van Zomeren et al., 2011). Participants indicated how often they had engaged in each of these activities in the past on 6-point scales ranging from “never,” “once,” “a few times,” “often,” “many times,” to “always.”

**Table 2.** Distribution of Answers to the Items Assessing Past Support for Social Change.

Item	Relative frequency (proportion) of answers provided by participants					
How often have you engaged in the following activities in the past?	Never	Once	A few times	Often	Many times	Always
1. Shared posts on Facebook or other social networks to support [DG’s] equality	.68	.07	.13	.06	.04	.02
2. Signed an online/regular petition to support action against the unequal treatment of [DG]	.73	.1	.1	.04	.02	.01
3. Wrote letters to public officials or other people of influence to protest against the unequal treatment of [DG]	.95	.03	.02	.01	.00	.00
4. Attended meetings or workshops regarding the unequal treatment of [DG]	.78	.08	.09	.03	.01	.00
5. Attended demonstrations, protests or rallies against the unequal treatment of [DG]	.86	.06	.05	.02	.01	.01
6. Voted for political candidates who support the equal treatment of [DG]	.55	.09	.13	.07	.06	.1

*Note.* Appropriate names for the disadvantaged group were inserted in each context. DG = disadvantaged group.

##### Intended support for social change

The items for intended support for social change (see Table 3) were identical to those used for past support except for the introductory sentence: “Would you like to engage in the following activities in the future?” Scales ranged from 1 = *Not at all* to 7 = *Very much*.

**Table 3.** Distribution of Answers to the Items Assessing Intended Support for Social Change.

Item	Relative frequency (proportion) of answers provided by participants						
Would you like to engage in the following activities in the future?	1 = *Not at all*	2	3	4	5	6	7 = *Very much*
1. Sharing posts on Facebook or other social networks to support [DG’s] equality	.32	.08	.07	.10	.12	.10	.20
2. Signing an online/regular petition to support action against the unequal treatment of [DG]	.33	.09	.08	.11	.12	.10	.17
3. Writing letters to public officials or other people of influence to protest against the unequal treatment of [DG]	.49	.17	.10	.11	.06	.03	.05
4. Attending meetings or workshops regarding the unequal treatment of [DG]	.36	.15	.12	.13	.12	.05	.08
5. Attending demonstrations, protests or rallies against the unequal treatment of [DG]	.43	.15	.09	.11	.09	.06	.06
6. Voting for political candidates who support the equal treatment of [DG]	.25	.08	.07	.12	.13	.12	.23

*Note.* Appropriate names for the disadvantaged group were inserted in each context. DG = disadvantaged group.

##### Positive contact

Two items assessed the extent to which advantaged group members experienced the contact with the disadvantaged group as positive (adapted from Tropp & Pettigrew, 2005): “When you interact with the outgroup, to what extent do you experience the following?” [“The contact is friendly,” “The contact is positive”]. Scales ranged from 1 = *Strongly disagree* to 7 = *Strongly agree*.

#### Data preparation

##### Accounting for the multilevel structure of the data

Following our preregistered plan, we group-mean-centered all predictors on the subsample level by subtracting the subsample means. By doing so, we removed the between-sample variance in the means, which allows us to focus on the fixed effects of the predictors in the following analyses (Bell et al., 2018, 2019).

##### Building measurement scales

We built scales based on the results of the Confirmatory Factor Analyses (CFA) following recommendations by Hu and Bentler (1999; i.e., Comparative Fit Index [CFI] of .95 or above, root mean square error of approximation [RMSEA] of .06 or less, and Standardized Root Mean Square Residual [SRMR] of less than .08) for all contact and support for social change variables. The fit was satisfactory for accepting contact and positive contact. Spearman-Brown correlations were .62 for the two items measuring accepting contact, and .89 for the two items measuring positive contact. Yet, CFA revealed an unsatisfactory fit for both past support for social change, where RMSEA (.09) was above the cutoff, and intended support for social change, where again RMSEA (.159) was above the cutoff. Modification indices suggested that fit could be improved by including only four items (attending meetings and workshops, signing petitions, sharing posts, and voting for political candidates) in the scales of both past support and intended support. Thus, we used these four item scales for all hypothesis tests and exploratory analyses. Cronbach’s alpha was .74 for past support and .87 for intended support for social change.

We suspect that the items about writing letters to public officials and attending demonstrations failed to load on the same factor as the four remaining items of support for social change because writing letters to public officials might not be a common practice among our participants, and demonstrations for the rights of the specific disadvantaged group may not have taken place in participants’ regions or participants may not have been aware of such events. Comparing the relative frequencies of answers given to the items of both past support (see Table 2) and intended support for social change (see Table 3) supports this reasoning: The frequency of participants who reported never having been involved or not intending to engage in either of the two actions was higher than for the other four items.

## Results

Descriptive statistics (means, standard deviations, and correlations) are presented in Table 4.

**Table 4.** Means, Standard Deviations, and Correlations for All Variables.

Variable	*M*	*SD*	1	2	3	4	5	6	7
1. Accepting contact	5.71	1.27							
2. Dual identity	4.52	1.90	.15**						
3. Common-ingroup identity	3.14	1.80	.27**	−.04*					
4. Separate identity	4.37	1.90	−.26**	.08**	−.60**				
5. Separate individuals	4.71	2.11	.12**	.07**	.24**	−.21**			
6. Past support for social change	1.77	0.97	.20**	.10**	.11**	−.10**	.07**		
7. Intended support for social change	3.58	1.89	.34**	.15**	.17**	−.15**	.11**	.57**	
8. Positive contact	5.46	1.40	.68**	.16**	.26**	−.24**	.11**	.23**	.38**

*Note. M* and *SD* represent mean and standard deviation, respectively, of the raw data values (i.e., before group-mean centering). The correlations are based on group-mean centered values.

* *p* < .05. **p < .01.

### Past Support for Social Change (Preregistered Hypothesis Tests)

**Figure 1.**
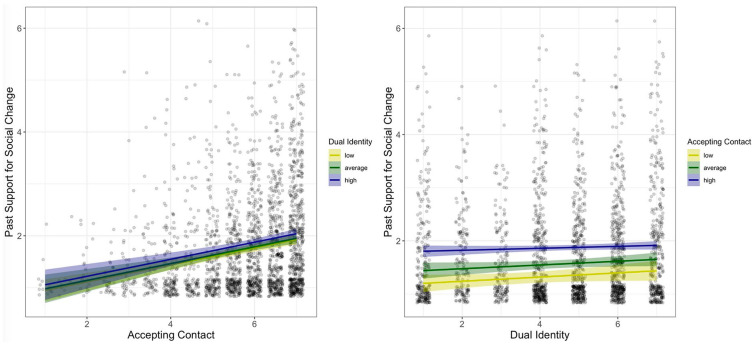
The left plot shows the relation between accepting contact and past support for social change under a low, average, and high dual identity. The right plot shows the relation between dual identity and past support for social change under low, average, and high levels of accepting contact. *Note.* Both plots show raw data points (with 80% transparency and overlapping data points depicted side by side) as well as regression lines and their corresponding 95% confidence regions.

We tested our hypotheses by regressing accepting contact, dual identity, and their interaction on past support for social change (see “core model” in Table 1). As predicted in H1 and H2, we found main effects of accepting contact, *b* = 0.17, *t*(2292) = 8.88, *p* < .001, β = .19, 95% CI^4^ = [0.15, 0.23], and dual identity, *b* = 0.07, *t*(2292) = 3.65, *p* < .001, β = .08, 95% CI = [0.03, 0.12], on past support for social change, such that more accepting contact as well as a stronger dual identity were associated with more past support for social change. These main effects were robust after omitting the interaction term from the regression model (for a full report of analyses, see online appendix). Contrary to H3, the interaction of accepting contact and dual identity was not significant, *b* = 0.02, *t*(2292) = 0.47, *p* = .635, β = .01, 95% CI = [−0.03, 0.04]; see Figure 1 for the obtained pattern of results.

### Intended Support for Social Change

**Figure 2.**
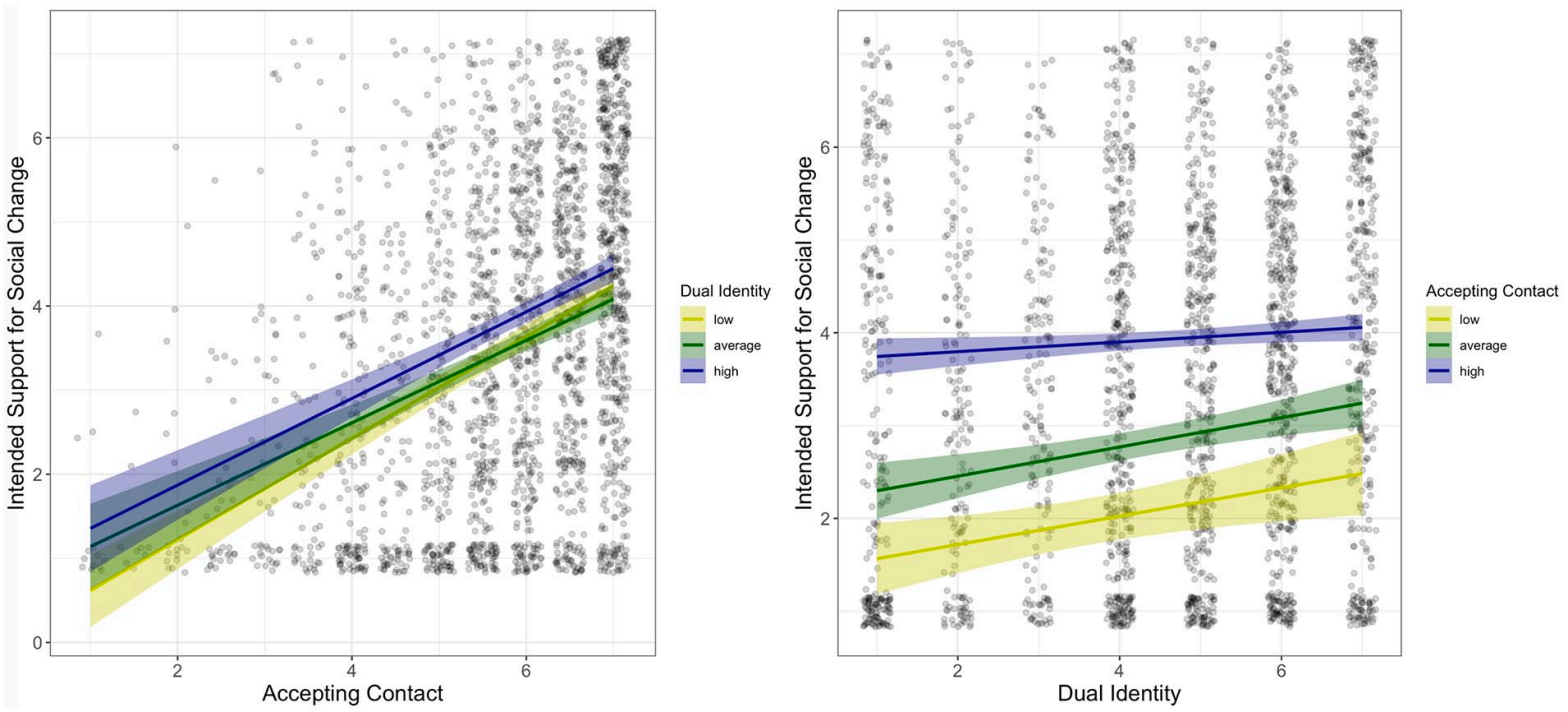
The left plot shows the relation between accepting contact and intended support for social change under a low, average, and high dual identity. The right plot shows the relation between dual identity and intended support for social change under low, average, and high levels of accepting contact. *Note.* Both plots show raw data points (with 80% transparency and overlapping data points depicted side by side) as well as regression lines and their corresponding 95% confidence regions.

Reflecting the gap between people’s behavioral intentions and actual behavior, participants indicated considerably higher levels of support for equality when asked about their future intentions as compared to their past behavior (compare Tables 2 and 3). Nevertheless, the pattern of results for intended support for social change as dependent variable, which is depicted in Figure 2, was similar to the pattern observed for past behavior. Analyses of the “core model” (see Table 1) yielded positive main effects of both accepting contact, *b* = 0.56, *t*(2287) = 16.03, *p* < .001, β = .32, 95% CI = [0.28, 0.36], and dual identity, *b* = 0.17, *t*(2287) = 5.06, *p* < .001, β = 0.1, 95% CI = [0.06, 0.14], but again there was no evidence for their interaction, *b* = −0.01, *t*(2287) = −0.37, *p* = .709, β = −.01, 95% CI = [−0.04, 0.03]. Again, the main effects of accepting contact and dual identity were robust after omitting the interaction term from the regression model (for a full report of analyses, see online appendix).

### Controlling for Covariates

Next, we examined the effects of accepting contact and dual identity after adding positive contact and the remaining identity representations (separate identity, common-ingroup identity, and separate individuals) as covariates in the two regression models, including all two-way interactions (following the recommendations by Yzerbyt et al., 2004; see Table 1 for an explication of model terms). Consistent with the above findings, analyses yielded positive main effects of accepting contact on past support, *b* = 0.06, *t*(2269) = 2.13, *p* = .033, β = .06, 95% CI = [0.01, 0.12], and on intended support for social change, *b* = 0.24, *t*(2264) = 4.92, *p* < .001, β = .14, 95% CI = [0.08, 0.19]. Thus, the effect of experiencing morally accepting intergroup contact on support for social change went beyond the effect of a mere positive contact experience. Furthermore, the positive main effects of dual identity on both past support, *b* = 0.07, *t*(2269) = 3.52, *p* < .001, β = .08, 95% CI = [0.03, 0.12], and intended support for social change, *b* = 0.15, *t*(2264) = 4.34, *p* < .001, β = .09, 95% CI = [0.05, 0.13], persisted. Thus, the effect of dual identity on support for social change went beyond the effects of the other types of representations. Finally, the interaction of accepting contact and dual identity was again nonsignificant for both past support, *b* = 0.03, *t*(2269) = 1.08, *p* = .279, β = .03, 95% CI = [−0.02, 0.08], and intended support for social change, *b* = 0.03, *t*(2264) = 0.72, *p* = .47, β = .02, 95% CI = [−0.03, 0.07].

Inspecting the covariates of the regression models (for a full report of analyses, see online appendix) revealed that common-ingroup identity, separate identity, and separate individuals had no significant main effects on past support (yet common-ingroup identity and separate individuals predicted more intended support). These findings are compatible with the common-ingroup identity model’s theorizing that a dual identity is the most beneficial identity representation in fostering support for social change among advantaged groups (Banfield & Dovidio, 2013). Moreover, neither a common-ingroup identity nor a separate identity, or separate individuals significantly interacted with accepting contact. These nonsignificant interactions suggest a robust positive relation between accepting contact and support for social change, regardless of the identity representations advantaged group members held.

Adding covariates such as age, gender, socioeconomic status, and ideology to the regression models or analyzing the effect of accepting contact and dual identity on support for social change within the single subsamples did not change our statistical conclusions (for a full report of the analyses, see online appendix).

### Post Hoc Exploration of the Hypotheses: Ordinal Bayesian Analyses

A closer examination of the data reveals three shortcomings of the preregistered analyses as reported above. Distributions of both support for social change variables are heavily skewed to the right. In fact, the most frequent answer participants gave was that they were “never” involved in a given action (see Table 2) and that they “not at all” intend to engage in this action in the future (see Table 3). Moreover, averaging across the items does not account for the possibility that people can share the same goal of intergroup equality but have divergent views on what is the best means to this end (see Craig et al., 2020; Louis et al., 2019; Sweetman et al., 2013). For instance, a participant who always attends workshops and meetings but does not engage in any other action to support social change has a lower average value of past support for social change than a participant with low to moderate past involvement in every single action. Finally, we cannot be certain that both measures of support for social change satisfied the assumptions of interval scaling (see Liddell & Kruschke, 2018).

To address these shortcomings, we created a new variable that contained for each participant only the maximum involvement in any kind of action supporting social change. By doing so, we test whether the maximum frequency of support for social change, regardless of the specific kind of action, can be predicted by accepting contact and a dual identity (and their interaction). This approach has three benefits: (a) we account for the possibility that not every action may have been a realistic option for participants (e.g., some participants were simply too young to have had the opportunity to vote for an equality endorsing candidate), (b) all six support for social change items can be used (rather than just the four items that remained following the factor analysis), and (c) we preserve the ordinal structure of both support for social change variables, which allows us to specify a more appropriate distribution of both variables.

We fitted Bayesian Generalized Multilevel Models (using the R-Package brms; Bürkner, 2017) by regressing the dependent variables (maximum past support for social change and maximum intended support for social change) on accepting contact, dual identity, and their interaction, allowing random intercepts and random slopes for each subsample. To account for the ordinal structure of the dependent variable, we used a cumulative link function. To improve model convergence, we specified minimally informative normal priors for all regression coefficients. Small R-hats (<1.01) and visual examinations of the trace plots showed satisfying model convergence.

The results suggested the same conclusions as the previous analyses. Specifically, inspecting the Highest Posterior Density Intervals (HPDIs), which give the range of the most credible values for an estimate, revealed that 93% of posterior estimates of accepting contact exceeded .3, while 81% of the posterior estimates of dual identity exceeded .1. The same pattern emerged when examining intended support for social change, where 95% of posterior estimates of accepting contact exceeded .5, and 91% of posterior estimates of dual identity exceeded .1. However, posterior estimates of the interaction terms varied closely around zero for past support (95% HPDI = [−0.06, 0.01]) and intended support for social change (95% HPDI = [−0.05, 0.01]; for full report of results, see online appendix).

Subsequent comparisons of the models that include versus exclude the interaction term (see Table 1 for an explication of model terms) based on the expected log pointwise density using approximate leave-one-out cross-validation (Vehtari et al., 2017) revealed a virtually identical fit. This suggests that, even if there were an interaction of accepting contact and dual identity, it would probably not systematically lead to better predictions of support for social change and, therefore, might be considered as practically negligible. Because of the virtually equivalent model performance, we tentatively favor the more parsimonious model, that is, the model without the interaction term. Thus, analyses based on the maximum frequency support for social change variables confirmed findings from the preregistered analyses, thereby increasing our confidence in the overall conclusion that accepting contact and dual identity *independently of one another* are associated with advantaged group members’ increased support for social change toward greater equality.

## Discussion

The present research replicates and extends previous research on the needs-based model (Shnabel & Nadler, 2015) and the common-ingroup identity model (Gaertner & Dovidio, 2000, 2012). We confirmed previous research by showing that both the experience of accepting contact (Hässler et al., 2020) and the endorsement of a dual identity representation of intergroup relations (Banfield & Dovidio, 2013) positively related to advantaged group members’ support for social change. In light of the increasing awareness of the importance of replications in the social sciences in general (e.g., Schmidt, 2009) and social psychology in particular (e.g., Nosek et al., 2015), these findings enhance the credibility of two prominent social psychological models. Moreover, using a large and diverse sample (note that Banfield and Dovidio’s, 2013, conclusions about the benefits of dual identity were based on relatively small samples of White U.S. participants), our findings extend the generalizability of these models (see also Eronen and Bringmann’s, 2021, call to gather more robust evidence for phenomena that are already “discovered” in psychology).

Unexpectedly, the results did not support our hypothesis that the positive effect of accepting contact will be particularly pronounced among advantaged group members who endorse a dual identity representation. Because our design allowed us to detect very small effects, this finding is unlikely to be due to low power. Moreover, model comparisons of Bayesian analyses suggested that the interaction term may be negligible. Therefore, our results are compatible with the idea that (a) the positive effect of accepting contact does not systematically depend on a dual identity representation, and (b) the positive effect of a dual identity representation does not systematically depend on accepting contact. Thus, experiencing the contact with disadvantaged group members as morally accepting positively related to support for social change among advantaged group members, regardless of whether the latter represent both groups in terms of a dual identity. Likewise, a dual identity representation was related to more support for equality among advantaged group members, regardless of whether contact with the disadvantaged group was experienced as accepting.

Notably, these results are robust across different types of analyses. First, our results were consistent for both past and intended support for social change. Yet, the level of support for social change was much higher when we asked advantaged group members about their future intentions, as compared with their actual behavior in the past. This finding is consistent with the idea of an intention–behavior gap (e.g., Webb & Sheeran, 2006; see also Dixon et al., 2017). Second, exploratory analyses showed that the positive relations between accepting contact and a dual identity representation on support for social change go beyond the effects of positive contact per se and the remaining identity representations (common-ingroup identity, separate identity, and separate individuals). And finally, Bayesian analyses that accounted for the ordinal scales and was based on all six support for social change items confirmed the results of the preregistered regression models, showing positive and independent relations of both dual identity and accepting contact with support for social change. We included these additional analyses because we acknowledge the impact researchers’ degrees of freedom may have on the results (see Simonsohn et al., 2020). While we preregistered our main analyses, it is still conceivable that the results were merely an artifact of some specific analytic decisions. Our Bayesian analyses differed from our preregistered analysis in several key points and led to qualitatively equivalent conclusions. This robustness of findings strengthens our confidence that both the common-ingroup identity model (Gaertner & Dovidio, 2000) and the needs-based model (Shnabel & Nadler, 2015) can be used independently of each other to predict support for social change among advantaged group members.

### Implications for Common-Ingroup Identity Model and Needs-Based Model

The present research has several implications for both theory and practice. By explicitly relating the common-ingroup identity model (Gaertner & Dovidio, 2000) to the needs-based model (Shnabel & Nadler, 2015), we were able to investigate potential boundary conditions of both models. The result that both models (i.e., accepting contact and a dual identity) predicted support for social equality independent of one another is good news for scholars and practitioners alike, which we elaborate in the following.

Starting with implications for the common-ingroup identity model, advantaged group members’ dual identity is associated with greater support for social equality, irrespective of their contact experiences with the disadvantaged group. This result provides empirical support to the theoretical frameworks proposed by Craig et al. (2020) and Radke et al. (2020) by underlining the importance of identity-related processes in spurring advantaged group members’ support for change. Furthermore, the examination of covariates supports the conclusion that a dual identity representation relates to advantaged group members’ support for social change, beyond any other identity representations. If a dual identity representation was simply the result of having both a high common-ingroup identity as well as high separate identity, a dual identity representation should not explain any variance in support for social change beyond what the combination of the other two identity representations is able to explain. Thus, our results suggest that the measure of a dual identity representation, namely, the ability to view both the commonalities and the differences between the groups at the same time, captured something extra—beyond the mere presence of both a common-ingroup identity and separate identity. For example, it may reflect the use of more complex cognitive processes and schemas (see, for example, Roccas and Brewer’s, 2002, notion of social identity complexity, which has been found to predict greater outgroup tolerance; Brewer & Pierce, 2005). This goes beyond previous research in which the logical relations between the measures of different identity representations have not been empirically examined. While the present study offers a starting point, further research is needed to better understand what exactly this “something extra” captures.

With regard to the needs-based model (Shnabel & Nadler, 2015), the present research confirms the finding that morally accepting contact with disadvantaged group members does not lead to the legitimization of intergroup inequalities (i.e., moral licensing effects; Merritt et al., 2010) but rather to endeavors aiming to reduce illegitimate inequalities (see Lowery et al., 2007, for conceptually consistent findings). This effect of accepting contact goes beyond the effect of mere positive contact: Advantaged group members’ support for social change depends on the satisfaction of their particular need to feel accepted and not morally condemned (Shnabel & Nadler, 2015; see also Lowery et al., 2007, for how affirming [vs. threatening] White participants’ moral identity increased their support for redistributive social policies).

In addition, by testing both models together for the first time, our findings shed light on the amount of support for social change that accepting contact and endorsement of a dual identity representation can explain on the population level (see Cumming’s, 2013, recommendation to pay greater attention to estimation based on effect sizes and confidence intervals in social psychological research). As such, they underscore the potential importance of intergroup contact for promoting group-based equality, suggesting that practitioners who design interventions to promote advantaged group members’ support for change should focus on creating opportunities for accepting contact (while making sure that it is also empowering for the disadvantaged group; Hässler et al., 2021), whereas targeting group members’ cognitive representations might be less crucial.

Finally, as our hypothesis pertaining to the interaction between accepting contact and dual identity on social change was not supported by the present data, we speculate on whether the interaction hypothesis might be supported with other methods, contexts, or conceptualizations of our key variables (following Simons et al.’s, 2017, recommendation to identify the boundary condition of reported effects). First, in line with the logic of the common-ingroup identity model, the wording of the identity representations items cued the identity of individuals living in the same country (e.g., Germany) as the superordinate category. However, the most inclusive meaningful superordinate category might be humanity. Possibly, had our measure cued a dual identity representation in which the separate categories refer to national, ethnic, and religious groups, yet the superordinate category refers to humanity—the expected moderation effect of dual identity and accepting contact on support for social change would have been found. This possibility is consistent with findings that human- versus national-level categorization can induce a greater sense of shared moral community (Wohl & Branscombe, 2005).

Second, it is plausible that, in societies that formally endorse egalitarian values, advantaged group members are generally concerned about their moral image in the eyes of the disadvantaged group, even in the absence of sharing a common superordinate identity. This is because disadvantaged outgroups are perceived as victims, due to a historic shift in the representation of minorities from morally deviant to victimized (Moscovici & Pérez, 2009). Therefore, accepting contact in such societies could be associated with social change behavior irrespective of identity representations. This finding broadens the generalizability of moral acceptance as a mechanism to facilitate social equality within societies advocating egalitarian values. However, it could be the case that moral acceptance does not increase advantaged group members’ support for social equality in societies in which minorities are represented as morally deviant rather than as victimized (Moscovici & Pérez, 2009) and egalitarianism is not formally endorsed (e.g., caste-based societies)—unless a dual identity representation is endorsed. This possibility should be tested in future research.

Third, while data were collected across several intergroup contexts and countries, areas characterized by extreme ongoing conflicts are underrepresented. It is plausible that dual identity does not affect the association between accepting contact and social change in contexts characterized mainly by structural inequality. However, a dual identity representation might facilitate the effect of accepting contact on social change to emerge in contexts characterized by direct forms of violence (see Galtung’s, 1969, distinction between structural and direct violence), where disadvantaged outgroups are at higher risk of exclusion from the moral community by advantaged group members.

### Limitations

The main limitation of the present study is that it is based on a cross-sectional survey, which limits causal inference. For example, while accepting contact and dual identity representation may increase support for social change, it is also possible that participation in activities (e.g., a demonstration or workshop) whose purpose is to promote social equality influences advantaged group members’ representation of intergroup relations and provides them an opportunity to experience accepting contact. This limitation is also relevant to our interpretation of the dependent variables. In the present research, we treated past behavior and behavioral intentions as two separate psychological outcomes. However, these two outcomes probably influence each other over time (e.g., intentions may eventually translate into actual behavior, and actual behavior may influence one’s future intentions^5^). Prospective, longitudinal studies may further clarify how exactly intentions relate to behavior in the context of support for social change and whether they can be predicted by earlier accepting contact and a dual identity representation.

Another limitation might be seen in the distribution of past support for social change, which was skewed to the right. However, it could be argued that the fact that past support was so infrequent makes reports of *any* past engagement in social change potentially highly informative. Having found associations between accepting contact and dual identity, on one hand, and past support, on the other hand, our research suggests that acting on accepting contact and dual identity might increase behavior aimed at social change.

Finally, although a major strength of our research is its generalizability across diverse samples and different types of measures of support for social change (i.e., past behavior and future intentions), we acknowledge that our findings are based on single operationalization of accepting contact and dual identity. Thus, the finding of the nonsignificant interaction of accepting contact and dual identity is tied to the specific measures we used to assess these variables. Future research is needed to assess the external validity of our results across different operationalizations (e.g., by manipulating accepting contact and identity representations).

## Conclusion

The present research extends the applicability of both the common-ingroup identity model and the needs-based model by showing that one model does not restrict the other, at least with regard to the associations with support for social change. Accepting contact and a dual identity representation related to advantaged group members’ support for social change, without conditioning one another. Although the lack of interaction was unexpected, refuting hypotheses is an important part of scientific advancement (Eronen & Bringmann, 2021). Importantly, our findings augment the field’s currently limited knowledge about precursors for advantaged groups’ support for intergroup equality. By doing so, the results have important implications for policymakers and practitioners in their effort to promote greater social equality.

## Supplemental Material

sj-docx-1-psp-10.1177_01461672221086380 – Supplemental material for Support for Social Change Among Members of Advantaged Groups: The Role of a Dual Identity Representation and Accepting Intergroup Contact Supplemental material, sj-docx-1-psp-10.1177_01461672221086380 for Support for Social Change Among Members of Advantaged Groups: The Role of a Dual Identity Representation and Accepting Intergroup Contact by Lisa Katharina Frisch, Simone Sebben, Luisa Liekefett, Nurit Shnabel, Emilio Paolo Visintin, Johannes Ullrich and Tabea Hässler in Personality and Social Psychology Bulletin

## Notes

1. When using intended support for social change as a dependent variable, Hässler et al. (2020, 2021) differentiated between high and low cost actions (e.g., attending a demonstration vs. sharing a post in the social networks, respectively). In the present study, however, we did not differentiate between high and low cost actions because factor analyses for the data we used suggested a one factor solution, for both past and intended support for social change.

2. As a consequence of this exclusion criteria, participants who reported to have no contact with members of the disadvantaged group are automatically excluded from the data set because of the questionnaire design: Participants who reported no contact were not presented any contact items which automatically results in more than 20% missing values (for two participants, the questionnaire filter did not work; both were excluded because of the attention check criterion).

3. In Hässler et al. (2020, 2021), accepting contact was measured on both the level of individuals and groups (e.g., “contact with outgroup left me with the impression that *my ingroup* is welcomed and accepted by outgroup members”). In the current article, we only focus on accepting contact on the level of individuals to be consistent with identity representation measures, which concern the individual level.

4. All confidence intervals refer to standardized effect sizes (β-weights).

5. See online appendix for a model in which past support was included as a predictor (besides the “core model” predictors) of future intentions; our key statistical conclusions remained unchanged.
