# Supplementary material for: Support for Social Change Among Members of Advantaged Groups: The Role of a Dual Identity Representation and Accepting Intergroup Contact
Source: Pers Soc Psychol Bull. 2022 Apr 28;49(7):1000–13. doi: 10.1177/01461672221086380 (PMC10302371; doi:10.1177/01461672221086380)
Supplement: sj-docx-1-psp-10.1177_01461672221086380 – Supplemental material for Support for Social Change Among Members of Advantaged Groups: The Role of a Dual Identity Representation and Accepting Intergroup Contact [file sj-docx-1-psp-10.1177_01461672221086380.docx]

**Appendix for**

Support for Social Change Among Members of Advantaged Groups: The Role of a Dual Identity Representation and Accepting Intergroup Contact

**OSF Project:** <https://osf.io/teb8g/?view_only=6701e6fc3c5749358db744a3c83949a6>

Table of Contents

[Samples 4](#_Toc92446220)

[Robustness Checks and Additional Analyses 5](#_Toc92446221)

*Core Model Without Interaction Term............................................................................*7

*Core Model with Different Exclusion Criteria...............................................................*8

*Controlling for Positive Contact and the Remaining Identity Representations............*11

*Controlling for Past Support when Predicting Intended Support for Social Change...*13

*Controlling for Demographic and Psychological Indicators.....................................*..14

*Subsample Analyses..................................................................................................*....16

*Post-Hoc Exploration of Hypotheses: Ordinal Bayesian Analyses..............................*17

**List of Tables**

[Table S1 Overview of Samples. 4](#_Toc38034714)

[Table S2 Model terms used in the robustness checks and additional analyses………………...6](#_Toc38034714)

[Table S3 Regression results on past support after omitting the interaction term 7](#_Toc38034714)

[Table S4 Regression results on intended support after omitting the interaction term 8](#_Toc38034714)

[Table S5 Regression results on past support using the full sample (no exclusions) 9](#_Toc38034714)

[Table S6 Regression results on intended support using the full sample (no exclusions) 9](#_Toc38034714)

[Table S7 Regression results on past support when administering the preregistered exclusion criteria only 10](#_Toc38034714)

[Table S8 Regression results on intended support when administering the preregistered exclusion criteria only 10](#_Toc38034714)

[Table S9 Regression results on past support when controlling for positive contact and the remaining identity representations. 11](#_Toc38034714)

[Table S10 Regression results on intended support when controlling for positive contact and the remaining identity representations. 12](#_Toc38034714)

[Table S11 Regression results on intended support when controlling for past support 14](#_Toc38034714)

[Table S12 Ordinal Bayesian Regression Results for Maximum Past Support for Social Change (Including the Interaction of Dual Identity and Accepting Contact). 18](#_Toc38034714)

[Table S13 Ordinal Bayesian Regression Results for Maximum Past Support for Social Change (Excluding the Interaction of Dual Identity and Accepting Contact) 18](#_Toc38034714)

[Table S14 Ordinal Bayesian Regression Results for Maximum Intended Support for Social Change (Including the Interaction of Dual Identity and Accepting Contact). 19](#_Toc38034715)

[Table S15 Ordinal Bayesian Regression Results for Maximum Intended Support for Social Change (Excluding the Interaction of Dual Identity and Accepting Contact) 19](#_Toc38034716)

[Table S16 Results of model comparisons for maximum past support for social change. 20](#_Toc38034717)

[Table S17 Results of model comparisons for maximum intended support for social change.. 20](#_Toc38034718)

# Samples

The overall sample includes 20 subsamples of racial, ethnic, and religious advantaged group members from 12 different countries. More information on the subsamples is given in Table S1. We focused on subsamples of racial, ethnic, and religious advantaged groups (and did not include LGBTQ+ samples, as done in previous work that used the ZIP dataset; e.g., Hässler et al., 2020).

Table S1

*Overview of Samples*

| Advantaged/  Disadvantaged Group | Country | *N* | Mean Age (*SD*) | Gender |  |  |
| --- | --- | --- | --- | --- | --- | --- |
| Belgians / Moroccans | Belgium | 65 | 18 (*2.65*) | male = 0, female = 65, other = 0 | | |
| Belgians / Turks | Belgium | 19 | 18 (*0.51*) | male = 0, female = 19, other = 0 | | |
| Whites / Blacks | Brazil | 155 | 28 (*12.01*) | male = 28, female = 126, other = 1 | | |
| Non- Indigenous / Mapuche | Chile | 128 | 26 (*10.25*) | male = 42, female = 86, other = 0 | | |
| Chileans / Peruvians | Chile | 120 | 23 (*14.23*) | male = 55, female = 65, other = 0 | | |
| Non- Muslims / Muslims | Germany | 158 | 23 (*11.37*) | male = 53, female = 102, other = 3 | | |
| Germans / Refugees | Germany | 117 | 23 (*12.31*) | male = 30, female = 85, other = 2 | | |
| Germans / Refugees | Germany | 137 | 34 (*12.18*) | male = 75, female = 62, other = 0 | | |
| Germans / Turks | Germany | 156 | 33 (*12.5*) | male = 86, female = 70, other = 0 | | |
| Jews / Arabs | Israel | 109 | 24 (*2.44*) | male = 32, female = 77, other = 0 | | |
| Non-Ethiopian Jews / Ethiopian Jews | Israel | 90 | 25 (*4.93*) | male = 36, female = 54, other = 0 | | |
| Albanians / Serbs | Kosovo | 28 | 20 (*1.43*) | male = 4, female = 24, other = 0 | | |
| Polish / Ukrainians | Poland | 86 | 33 (*14.08*) | male = 37, female = 49, other = 0 | | |
| Serbs / Bosniaks | Serbia | 79 | 21 (*2.19*) | male = 15, female = 64, other = 0 | | |
| Spaniards / Sinti & Roma | Spain | 444 | 22 (*7.32*) | male = 140, female = 301, other = 3 | | |
| Non- Muslims / Muslims | Switzerland | 95 | 23 (*11.24*) | male = 30, female = 65, other = 0 | | |
| Swiss nationals/ Portuguese immigrants | Switzerland | 107 | 25 (*9.77*) | male = 26, female = 81, other = 0 | | |
| Non- Muslims / Muslims | United Kingdom | 123 | 33 (*11.26*) | male = 60, female = 62, other = 1 | | |
| Britons / Asians | United Kingdom | 85 | 19 (*5.85*) | male = 21, female = 64, other = 0 | | |
| Non- Muslims / Muslims | United States | 3 | 40 (*9.6*) | male = 3, female = 0, other = 0 | | |

*Note.* Information on subsamples is given after administering the exclusions that are described in the main text.

# Robustness Checks and Additional Analyses

In the following sections we report analyses testing whether our main findings depend on a) the exclusion of the interaction term from the core model, b) exclusion criteria, c) the inclusion of positive contact and the remaining identity representation as covariates, d) the inclusion of past support when predicting intended support for social change, e) the inclusion of demographic indicators and psychological variables as covariates, f) the different subsamples, and g) the type of analyses (i.e., Ordinal Bayesian Regressions). Table S2 provides an overview of the regression models tested. All results can be reproduced using the data and R code on OSF (<https://osf.io/teb8g/?view_only=6701e6fc3c5749358db744a3c83949a6>).

Table S2

*Model terms used in the robustness checks and additional analyses*

| Analysis | Model |
| --- | --- |
| 1. Core model without interaction term | Past/Intended support = Accepting contact + Dual identity |
| 1. Different exclusion criteria | Past/Intended support = Accepting contact × Dual identity |
| 1. Controlling for positive contact and remaining identity representation | Past/Intended support = Accepting contact × Dual identity × Positive contact × Common-ingroup identity × Separate identities × Separate individuals |
| 1. Controlling for past support when predicting intended support | Intended support = Accepting contact × Dual identity × Past support |
| 1. Controlling for demographic and psychological indicators   (64 models for each past and intended support) | Past/Intended support = Accepting contact × Dual identity  Past/Intended support = Accepting contact × Dual identity + Age  Past/Intended support = Accepting contact × Dual identity + Gender  Past/Intended support = Accepting contact × Dual identity + SES  Past/Intended support = Accepting contact × Dual identity + Ideology  Past/Intended support = Accepting contact × Dual identity + Age + Gender  Past/Intended support = Accepting contact × Dual identity + Age + SES  Past/Intended support = Accepting contact × Dual identity + Age + Ideology  Past/Intended support = Accepting contact × Dual identity + Gender + SES  Past/Intended support = Accepting contact × Dual identity + Gender + Ideology  Past/Intended support = Accepting contact × Dual identity + SES + Ideology  Past/Intended support = Accepting contact × Dual identity + Age + Gender + SES  Past/Intended support = Accepting contact × Dual identity + Age + Gender + Ideology  Past/Intended support = Accepting contact × Dual identity + Age + SES + Ideology  Past/Intended support = Accepting contact × Dual identity + Gender + SES + Ideolog  Past/Intended support = Accepting contact × Dual identity + age + gender + SES + Ideology  Past/Intended support = Accepting contact × Dual identity × Positive contact  …  Past/Intended support = Accepting contact × Dual identity × Positive contact + age + gender + SES + Ideology  Past/Intended support = Accepting contact × Dual identity × Common-ingroup identity × Separate identities × Separate individuals  …  Past/Intended support = Accepting contact × Dual identity × Common-ingroup identity × Separate identities × Separate individuals + age + gender + SES + Ideology  Past/Intended support = Accepting contact × Dual identity × Positive contact × Common-ingroup identity × Separate identities × Separate individuals  …  Past/Intended support = Accepting contact × Dual identity × Positive contact × Common-ingroup identity × Separate identities × Separate individuals + age + gender + SES + Ideology |
| 1. Subsample Analyses | Past/Intended support = Accepting contact × Dual identity |
| 1. Ordinal Bayesian Analyses | Past/Intended support = Accepting contact × Dual identity  Past/Intended support = Accepting contact + Dual identity |

*Note.* We ran each model for past support and intended support separately*.* × Indicates that main effects and two-way interactions were tested. + Indicates that main effects were tested. … Indicates that all possible combinations of demographic indicators were included into the model as main effects. Note that Ideology contains two variables, system justification and perceived legitimacy.

**Core Model Without Interaction Term**

As reported in the main text, the two positive main effects of accepting contact and dual identity on both past and intended support for social change did not depend on whether we included or excluded the interaction term from the regression model. We report the results of regression analyses omitting the interaction term in Table S3 for past support, and in Table S4 for intended support for social change.

Table S3

*Regression results on past support after omitting the interaction term*

| Predictor | *b* | ß | ß 95% CI  [LL, UL] | *p* |
| --- | --- | --- | --- | --- |
| (Intercept) | 0.00 | 0.00 | [-0.04, 0.04] | .982 |
| Accepting contact | 0.17 | 0.19 | [0.14, 0.23] | < .001 |
| Dual identity | 0.07 | 0.07 | [0.03, 0.12] | < .001 |

*Note.* *b* indicates the unstandardized regression coefficient, and *LL* and *UL* indicate the lower and upper limits of the 95% confidence interval of the standardized coefficient *ß*, respectively.

Table S4

*Regression results on intended support after omitting the interaction term.*

| Predictor | *b* | ß | ß 95% CI  [LL, UL] | *p* |
| --- | --- | --- | --- | --- |
| (Intercept) | 0.00 | 0.00 | [-0.04, 0.04] | .905 |
| Accepting contact | 0.56 | 0.33 | [0.29, 0.36] | < .001 |
| Dual identity | 0.17 | 0.10 | [0.06, 0.14] | < .001 |

*Note.* *b* indicates the unstandardized regression coefficient, and *LL* and *UL* indicate the lower and upper limits of the 95% confidence interval of the standardized coefficient *ß*, respectively.

**Different Exclusion Criteria**

The statistical conclusions we draw from the preregistered hypotheses tests (i.e., the core model, see Table S2) do not depend on whether we include or exclude participants based on the exclusion criteria. As described in the main text, we preregistered to exclude all participants that failed on one or both attention checks, and who had more than 20% missing values on the items used in the present paper. Not preregistered, but reasonable after seeing the data, we further excluded samples that were collected after the main wave of data collection, and samples that used sliders instead of Likert scales to assess support for social change.

Table S5 and S6 summarize the results from the core model for past and intended support for social change when using the full sample without any exclusions (*N* = 4,105). Table S7 and S8 summarize the results from the core model when administering only the preregistered (*N* = 2,496) and not the non-preregistered exclusion criteria used for the main analyses (*N* = 2,304). As apparent from Tables S5 – S8, our main findings of two main effects of accepting contact and dual identity were supported irrespective of the exclusion criteria. Again, we found no support for the interaction of accepting contact and dual identity. The only exception is Table S6, in which the interaction term has reached statistical significance. However, we do not consider this finding reliable evidence in support of the interaction because the effect failed to emerge systematically across different robustness analyses.

Table S5

*Regression results on past support using the full sample (no exclusions).*

| Predictor | *b* | ß | ß 95% CI  [LL, UL] | *p* |
| --- | --- | --- | --- | --- |
| (Intercept) | 1.80 | 0.00 | [-0.03, 0.04] | < .001 |
| Accepting contact | 0.14 | 0.14 | [0.10, 0.17] | < .001 |
| Dual identity | 0.07 | 0.07 | [0.03, 0.10] | < .001 |
| Accepting contact × Dual Identity | -0.02 | -0.02 | [-0.05, 0.02] | .319 |

*Note.* *b* indicates the unstandardized regression coefficient, and *LL* and *UL* indicate the lower and upper limits of the 95% confidence interval of the standardized coefficient *ß*, respectively.

Table S6

*Regression results on intended support using the full sample (no exclusions).*

| Predictor | *b* | ß | ß 95% CI  [LL, UL] | *p* |
| --- | --- | --- | --- | --- |
| (Intercept) | 3.56 | 0.00 | [-0.03, 0.04] | < .001 |
| Accepting contact | 0.62 | 0.33 | [0.30, 0.37] | < .001 |
| Dual identity | 0.18 | 0.09 | [0.06, 0.13] | < .001 |
| Accepting contact × Dual Identity | -0.06 | -0.03 | [-0.06, 0.01] | .023 |

*Note.* *b* indicates the unstandardized regression coefficient, and *LL* and *UL* indicate the lower and upper limits of the 95% confidence interval of the standardized coefficient *ß*, respectively.

Table S7

*Regression results on past support when administering the preregistered exclusion criteria only (N = 2496)*

| Predictor | *b* | ß | ß 95% CI  [LL, UL] | *p* |
| --- | --- | --- | --- | --- |
| (Intercept) | 0.00 | 0.00 | [-0.04, 0.04] | .094 |
| Accepting contact | 0.16 | 0.18 | [0.14, 0.22] | < .001 |
| Dual identity | 0.06 | 0.07 | [0.03, 0.11] | < .001 |
| Accepting contact × Dual Identity | 0.01 | 0.01 | [-0.03, 0.04] | .699 |

*Note.* *b* indicates the unstandardized regression coefficient, and *LL* and *UL* indicate the lower and upper limits of the 95% confidence interval of the standardized coefficient *ß*, respectively.

Table S8

*Regression results on intended support for social change when administering the preregistered exclusion criteria only (N = 2496)*

| Predictor | *b* | ß | ß 95% CI  [LL, UL] | *p* |
| --- | --- | --- | --- | --- |
| (Intercept) | 0.00 | 0.00 | [-0.03, 0.04] | .999 |
| Accepting contact | 0.54 | 0.32 | [0.30, 0.37] | < .001 |
| Dual identity | 0.15 | 0.09 | [0.06, 0.13] | < .001 |
| Accepting contact × Dual Identity | -0.02 | -0.01 | [-0.06, 0.00] | .513 |

*Note.* *b* indicates the unstandardized regression coefficient, and *LL* and *UL* indicate the lower and upper limits of the 95% confidence interval of the standardized coefficient *ß*, respectively.

# Controlling for Positive Contact and the Remaining Identity Representations

In this section we present the effects of accepting contact and dual identity after adding positive contact, and the remaining identity representations (separate identities, common-ingroup identity, and separate individuals) as covariates in the regression model, including all two-way interactions. Table S9 presents regression results for past support for social change. Table S10 presents regression results for intended support for social change. As can be seen in Tables S9 and S10, the effects reported in the main text remain significant after including these additional covariates.

Table S9

*Regression results on past support when controlling for positive contact and the remaining identity representations*

| Predictor | *b* | *ß* | *ß*  95% CI  [LL, UL] | *p* |
| --- | --- | --- | --- | --- |
| (Intercept) | -0.03 | -0.03 | [-0.08, 0.02] | .222 |
| Accepting contact | 0.06 | 0.06 | [ 0.01, 0.12] | .033 |
| Dual identity | 0.07 | 0.08 | [ 0.03, 0.12] | <.001 |
| Positive contact | 0.16 | 0.18 | [ 0.12, 0.24] | <.001 |
| Separate identities | -0.03 | -0.03 | [-0.08, 0.02] | .228 |
| Common-ingroup identity | 0.02 | 0.03 | [-0.03, 0.08] | .355 |
| Separate individuals | 0.02 | 0.02 | [-0.02, 0.06] | .302 |
| Accepting contact × Dual identity | 0.03 | 0.03 | [-0.02, 0.08] | .279 |
| Accepting contact × Positive contact | 0.03 | 0.04 | [ 0.00, 0.07] | .058 |
| Accepting contact × Separate identities | 0.00 | 0.00 | [-0.07, 0.07] | .975 |
| Accepting contact × Common-ingroup identity | -0.02 | -0.02 | [-0.09, 0.05] | .574 |
| Accepting contact × Separate individuals | 0.01 | 0.01 | [-0.04, 0.07] | .690 |
| Dual identity × Positive contact | -0.02 | -0.02 | [-0.07, 0.04] | .501 |
| Dual identity × Separate identities | -0.01 | -0.01 | [-0.06, 0.04] | .675 |
| Dual identity × Common-ingroup identity | -0.02 | -0.02 | [-0.07, 0.02] | .319 |
| Dual identity × Separate individuals | -0.01 | -0.01 | [-0.05, 0.02] | .468 |
| Positive contact × Separate identities | 0.02 | 0.02 | [-0.05, 0.08] | .589 |
| Positive contact × Common-ingroup identity | 0.03 | 0.03 | [-0.04, 0.10] | .365 |
| Positive contact × Separate individuals | -0.01 | -0.02 | [-0.07, 0.04] | .578 |
| Separate identities × Common-ingroup identity | -0.02 | -0.02 | [-0.06, 0.02] | .378 |
| Separate identities × Separate individuals | 0.02 | 0.02 | [-0.03, 0.07] | .411 |
| Common-ingroup identity  × Separate individuals | 0.01 | 0.01 | [-0.04, 0.06] | .737 |
|  |  |  |  |  |

*Note.* *b* indicates the unstandardized regression coefficient, and *LL* and *UL* indicate the lower and upper limits of the 95% confidence interval of the standardized coefficient *ß*, respectively.

Table S10

*Regression results on intended support when controlling for positive contact and the remaining identity representations*

| Predictor |  | *b* | *ß* | *ß*  95% CI  [LL, UL] | *p* |
| --- | --- | --- | --- | --- | --- |
| (Intercept) | | 0.04 | 0 | [-0.04, 0.05] | .913 |
| Accepting contact | | 0.24 | 0.14 | [ 0.08, 0.19] | < .001 |
| Dual identity | | 0.15 | 0.09 | [ 0.05, 0.13] | < .001 |
| Positive contact | | 0.42 | 0.25 | [ 0.20, 0.30] | < .001 |
| Separate identities | | -0.02 | -0.01 | [-0.06, 0.04] | .579 |
| Common-ingroup identity | | 0.09 | 0.05 | [ 0.00, 0.11] | .04 |
| Separate individuals | | 0.07 | 0.04 | [ 0.00, 0.08] | .031 |
| Accepting contact × Dual identity | | 0.03 | 0.02 | [-0.03, 0.07] | .47 |
| Accepting contact × Positive contact | | 0.02 | 0.01 | [-0.02, 0.05] | .538 |
| Accepting contact × Separate identities | | 0.05 | 0.03 | [-0.03, 0.10] | .339 |
| Accepting contact × Common-ingroup identity | | 0.05 | 0.03 | [-0.03, 0.10] | .345 |
| Accepting contact × Separate individuals | | 0.02 | 0.01 | [-0.04, 0.07] | .586 |
| Dual identity × Positive contact | | 0 | 0 | [-0.05, 0.05] | .961 |
| Dual identity × Separate identities | | 0 | 0 | [-0.04, 0.04] | .981 |
| Dual identity × Common-ingroup identity | | -0.02 | -0.01 | [-0.06, 0.03] | .633 |
| Dual identity × Separate individuals | | -0.08 | -0.05 | [-0.09, -0.01] | .012 |
| Positive contact × Separate identities | | 0.01 | 0 | [-0.06, 0.07] | .923 |
| Positive contact × Common-ingroup identity | | -0.02 | -0.01 | [-0.08, 0.05] | .715 |
| Positive contact × Separate individuals | | -0.02 | -0.01 | [-0.06, 0.04] | .665 |
| Separate identities × Common-ingroup identity | | 0 | 0 | [-0.04, 0.04] | .95 |
| Separate identities × Separate individuals | | 0.01 | 0.01 | [-0.04, 0.05] | .764 |
| Common-ingroup identity  × Separate individuals | | -0.04 | -0.02 | [-0.07, 0.02] | .299 |

*Note.* *b* indicates the unstandardized regression coefficient, and *LL* and *UL* indicate the lower and upper limits of the 95% confidence interval of the standardized coefficient *ß*, respectively.

**Controlling for Past Support When Predicting Intended Support for Social Change**

Since past behavior is a predictor of future intentions (Ouellette & Wood, 1998), there is reason to assume a correlation between past support and intended support for social change. In fact, their correlation in the present dataset was *r* = .57 (see Table 4 in the main text). Thus, controlling for reports of past behavior while predicting intended support for social change reveals how much dual identity and accepting contact contribute to people’s intentions to support social change beyond what can be predicted solely based on their past support for social change. We regressed intended support on accepting contact, dual identity, and past support, allowing all two-way interactions (see Table S2). Corroborating our prior results and as reported in Table S11, analyses revealed two main effects (such that both accepting contact and dual identity were related to greater intended support for social change) but no significant interaction between them. Furthermore, there was a positive main effect of past support for social change, such that more past support was related to stronger intentions to support social change in the future. There was no significant interaction of past support neither with accepting contact nor with dual identity. Thus, accepting contact and dual identity contribute to peoples’ intentions to support social change, above and beyond what can be predicted based on their past support for social change.

Table S11

*Regression results on intended support when controlling for past support*

| Predictor | *b* | ß | ß 95% CI  [LL, UL] | *p* |
| --- | --- | --- | --- | --- |
| (Intercept) | 0.01 | 0.01 | [-0.02, 0.04] | .651 |
| Accepting contact | 0.38 | 0.22 | [ 0.19, 0.25] | < .001 |
| Dual identity | 0.10 | 0.06 | [ 0.03, 0.09] | < .001 |
| Past support | 0.91 | 0.53 | [ 0.50, 0.57] | < .001 |
| Accepting contact × Dual Identity | -0.00 | 0.00 | [-0.03, 0.03] | .923 |
| Accepting contact × Past support | -0.06 | -0.03 | [-0.07, 0.00] | .051 |
| Dual Identity × Past support | -0.04 | -0.02 | [-0.06, 0.01] | .144 |

*Note.* *b* indicated the unstandardized regression coefficient, and *LL* and *UL* indicate the lower and upper limits of the 95% confidence interval of the standardized coefficient *ß*, respectively.

**Controlling for Demographic and Psychological Indicators**

We conducted regression analyses that tested whether our findings persist even when controlling for key demographic and psychological indicators; age, gender, socioeconomic status (SES), and ideology (consistent with previous work that used the ZIP dataset; Hässler et al., 2020, 2021). SES was assessed with the MacArthur SES ladder (Adler et al., 2000), which asks respondents to place themselves on a social ladder where the highest (lowest) rung represents individuals with the most (least) money, highest (lowest) education, and the best (worst) jobs in their society. Following Hässler, Ullrich et al. (2021), we used perceived legitimacy of intergroup contexts (2 items) and system justification (6 items) as measures of ideology.

We systematically varied the inclusion of all possible combinations of age, gender, SES, and ideology to

(a) the core model (including accepting contact, dual identity and their interaction as predictors; see Table S2),

(b) the core model and positive contact,

(c) the core model and the remaining identity representations (separate identity, common-ingroup identity, and separate individuals), and

(d) the core model including both positive contact and the remaining identity representations.

For all contact and identity representation variables we allowed two-way interactions; the demographic and psychological indicators were included without interactions. The two ideology measures (perceived legitimacy and system justification) were included as a set.

In total, we analyzed 16 (all combinations of demographic and psychological indicators) × 4 (models (a) – (d)) = 64 regression models for past support and intended support for social change each (see Table S2 for an explication of model terms). For both dependent variables, we inspected how often the models confirmed the main pattern of results as reported in the main text; namely, a positive main effect of accepting contact, a positive main effect of dual identity, and a non-significant interaction.

For past support for social change as the dependent variable, the vast majority of the 64 models confirmed the main pattern of results. Specifically, for accepting contact, the median coefficient was 0.1 (*M* = 0.11, *SD* = 0.05), with all coefficients being positive, and 88% being significant at *p* < .05. For dual identity, the median coefficient was 0.07 (*M* = 0.07, *SD* = 0.01), with 100% of coefficients being positive and significant at *p* < .05. The interaction of accepting contact and dual identity revealed a median coefficient of 0.02 (*M* = 0.02, *SD* = 0.01) with 0% of coefficients being statistically significant at *p* < .05.

Analyses revealed similar results for intended support for social change as the dependent variable. The median coefficient was 0.34 (*M* = 0.35, *SD* = 0.13) for accepting contact, and 0.15 (*M* = 0.15, *SD* = 0.02) for dual identity, with 100% of coefficients being positive and significant at *p* < .05. As for the interaction of accepting contact and dual identity, the median coefficient was 0.02 (*M* = 0.02, *SD* = 0.02) with 0% of coefficients being statistically significant at *p* < .05. For individual regression results the reader is referred to the R code, l. 554-800, on OSF (<https://osf.io/teb8g/?view_only=6701e6fc3c5749358db744a3c83949a6>).

To summarize, adding demographic and psychological covariates such as age, gender, SES and ideology to the regression models did not change our statistical conclusions about two main effects of accepting contact and dual identity on past and intended support for social change and their non-significant interaction.

**Subsample Analyses**

To examine whether the main pattern of results is robust in the subsamples, we regressed accepting contact, dual identity, and their interaction on both past and intended support for social change within each of the subsamples with *n* > 50. Three subsamples (i.e., USA/Muslims, Albanians/Serbs, Belgians/Turks) were excluded from the analyses due to a smaller sample size. Thus, the final number of subsamples was *n* = 17. As with the analyses of demographic and psychological indicators, we inspected whether the main pattern of results – positive coefficients for both accepting contact and dual identity, and coefficients ranging closely around zero for their interaction – was confirmed within the subsamples. We report the coefficients but refrain from reporting *p*-values because they lack interpretability due to the substantially smaller sample sizes and the resulting lack of statistical power.

For past support for social change as the dependent variable, the median coefficient for accepting contact was 0.15 (*M* = 0.13, *SD* = 0.13,), the median coefficient for dual identity was 0.05 (*M* = 0.05, *SD* = 0.13), and the median for their interaction was 0.01 (*M* = 0.02, *SD* = 0.06). For intended support for social change as the dependent variable, the pattern was similar: the median coefficient for accepting contact was 0.58 (*M* = 0.53, *SD* = 0.19), the median coefficient for dual identity was 0.11 (*M* = 0.11, *SD* = 0.21), and the median coefficient for their interaction was -0.01 (*M* = 0.02, *SD* = 0.18). Readers interested in the results for specific subsamples are kindly referred to the R code, l. 802-880, on OSF (<https://osf.io/teb8g/?view_only=6701e6fc3c5749358db744a3c83949a6>).

To summarize, the subsample analyses corroborated our conclusions about two additive positive effects of both accepting contact and dual identity on support for social change.

# Post-hoc Exploration of the Hypotheses: Ordinal Bayesian Analyses

In this section, we present full results of the ordinal Bayesian regression models examining maximum past and maximum intended support for social change. Table S12 and S13 show the results for past support for social change, with the interaction of dual identity and accepting contact included and excluded, respectively. Table S14 and S15 show the results for intended support for social change with the interaction term included vs. excluded, respectively. Please note that the intervals as reported in the Tables are Credible Intervals, and not Highest Posterior Density Intervals (HPDI). For more information on HPDIs we refer the reader to the R code, l. 381-477, on OSF (<https://osf.io/teb8g/?view_only=6701e6fc3c5749358db744a3c83949a6>).

Table S12

*Ordinal Bayesian Regression Results for Maximum Past Support for Social Change (Including the Interaction of Dual Identity and Accepting Contact)*

|  | Estimate | Est. Error | Lower 95% CI | Upper 95% CI | R-hat |
| --- | --- | --- | --- | --- | --- |
| Intercept[1] | 2.34 | 0.51 | 1.35 | 3.37 | 1.00 |
| Intercept[2] | 2.95 | 0.51 | 1.96 | 3.98 | 1.00 |
| Intercept[3] | 3.88 | 0.52 | 2.88 | 4.92 | 1.00 |
| Intercept[4] | 4.53 | 0.52 | 3.54 | 5.58 | 1.00 |
| Intercept[5] | 5.27 | 0.52 | 4.27 | 6.32 | 1.00 |
| Dual Identity | 0.19 | 0.1 | -0.01 | 0.38 | 1.00 |
| Accepting contact | 0.42 | 0.09 | 0.26 | 0.59 | 1.00 |
| Dual Identity × Accepting contact | -0.02 | 0.02 | -0.06 | 0.01 | 1.00 |

Table S13

*Ordinal Bayesian Regression Results for Maximum Past Support for Social Change (Excluding the Interaction of Dual Identity and Accepting Contact)*

|  | Estimate | Est. Error | Lower 95% CI | Upper 95% CI | R-hat |
| --- | --- | --- | --- | --- | --- |
| Intercept[1] | 1.78 | 0.33 | 1.13 | 2.43 | 1.00 |
| Intercept[2] | 2.39 | 0.33 | 1.74 | 3.04 | 1.00 |
| Intercept[3] | 3.32 | 0.33 | 2.66 | 3.98 | 1.00 |
| Intercept[4] | 3.97 | 0.33 | 3.31 | 4.64 | 1.00 |
| Intercept[5] | 4.71 | 0.34 | 4.03 | 5.38 | 1.00 |
| Dual Identity | 0.06 | 0.03 | -0.01 | 0.12 | 1.00 |
| Accepting contact | 0.33 | 0.05 | 0.22 | 0.43 | 1.00 |

Table S14

*Ordinal Bayesian Regression Results for Maximum Intended Support for Social Change (Including the Interaction of Dual Identity and Accepting Contact)*

|  | Estimate | Est. Error | Lower 95% CI | Upper 95% CI | R-hat |
| --- | --- | --- | --- | --- | --- |
| Intercept[1] | 2.36 | 0.48 | 1.43 | 3.28 | 1.00 |
| Intercept[2] | 2.75 | 0.48 | 1.82 | 3.67 | 1.00 |
| Intercept[3] | 3.18 | 0.48 | 2.25 | 4.11 | 1.00 |
| Intercept[4] | 3.68 | 0.48 | 2.75 | 4.61 | 1.00 |
| Intercept[5] | 4.37 | 0.48 | 3.43 | 5.31 | 1.00 |
| Intercept[6] | 5.1 | 0.48 | 4.17 | 6.04 | 1.00 |
| Dual Identity | 0.23 | 0.1 | 0.04 | 0.42 | 1.00 |
| Accepting contact | 0.63 | 0.08 | 0.48 | 0.79 | 1.00 |
| Dual Identity × Accepting contact | -0.02 | 0.02 | -0.05 | 0.01 | 1.00 |

Table S15

*Ordinal Bayesian Regression Results for Maximum Intended Support for Social Change (Excluding the Interaction of Dual Identity and Accepting Contact)*

|  | Estimate | Est. Error | Lower 95% CI | Upper 95% CI | R-hat |
| --- | --- | --- | --- | --- | --- |
| Intercept[1] | 1.83 | 0.29 | 1.27 | 2.39 | 1.00 |
| Intercept[2] | 2.22 | 0.29 | 1.65 | 2.79 | 1.00 |
| Intercept[3] | 2.65 | 0.29 | 2.07 | 3.23 | 1.00 |
| Intercept[4] | 3.15 | 0.29 | 2.58 | 3.73 | 1.00 |
| Intercept[5] | 3.84 | 0.3 | 3.26 | 4.43 | 1.00 |
| Intercept[6] | 4.57 | 0.3 | 3.98 | 5.17 | 1.00 |
| Dual Identity | 0.1 | 0.03 | 0.04 | 0.16 | 1.00 |
| Accepting contact | 0.54 | 0.04 | 0.46 | 0.61 | 1.00 |

Subsequent comparisons of models that include vs. exclude the interaction term based on the expected log pointwise density using approximate leave-one-out cross-validation, revealed a virtually identical fit (following suggestions by Vehtari et al., 2017). We present the results in Table S16 for maximum past support for social change and in Table S17 for maximum intended support for social change.

Table S16

*Results of model comparisons for maximum past support for social change.*

| Model | Difference | SE |
| --- | --- | --- |
| Model excluding the interaction | -0.3 | 1.6 |
| Model including the interaction | 0.0 | 0.0 |

Table S17

*Results of model comparisons for maximum intended support for social change.*

| Model | Difference | SE |
| --- | --- | --- |
| Model excluding the interaction | 0.0 | 0.0 |
| Model including the interaction | -0.4 | 1.7 |

**References**

Adler, N. E., Epel, E. S., Castellazzo, G., & Ickovics, J. R. (2000). Relationship of subjective and objective social status with psychological and physiological functioning: Preliminary data in healthy, White women. *Health Psychology, 19*(6), 586–592. <https://doi.org/10.1037/0278-6133.19.6.586>

Hässler, T., Ullrich, J., Bernadino, M., Shnabel, N., van Laar, C., Valdenegro, D., Sebben, S., Tropp, L. R., Visintin, E. P., González, R., Ditlmann, R.K., Abrams, D., Selvanathan, H. P., Branković, M., Wright, S., von Zimmermann, J., Pasek, M., Aydin, A. L., Žeželj, I., . . . Ugarte, L. M. (2020). A large-scale test of the link between intergroup contact and support for social change. *Nature Human Behaviour, 4*, 380–386. <https://doi.org/10.1038/s41562-019-0815-z>

Hässler, T., Ullrich, J., Sebben, S., Shnabel, N., Bernadino, M., Valdenegro, D., van Laar, C., González, R., Visintin, E. P., Tropp, L. R., Ditlmann, R. K., Abrams, D., Aydin, A. L., Pereira, A., Selvanathan, H. P., von Zimmermann, J., Lantos, N., Sainz, M. Glenz, A., . . .Pistella, J. (2021). Needs satisfaction in intergroup contact: A multi-national study of pathways toward social change. *Journal of Personality and Social Psychology*. Advance online publication. <https://doi.org/10.1037/pspi0000365>

Ouellette, J. A., & Wood, W. (1998). Habit and intention in everyday life: The multiple processes by which past behavior predicts future behavior. *Psychological Bulletin, 124*(1), 54–74. [https://doi.org/10.1037/0033-2909.124.1.54](https://psycnet.apa.org/doi/10.1037/0033-2909.124.1.54)

Vehtari, A., Gelman, A., & Gabry, J. (2017). Practical Bayesian model evaluation using leave-one-out cross-validation and WAIC. *Statistics and computing*, *27*(5), 1413-1432. doi: 10.1007/s11222-016-9696-4
